# P23. Efficient ex vivo lysis of acute myeloid leukaemic (AML) cells mediated by triplebodies with dual-targeting capability in conjunction with natural killer cells as effectors

**DOI:** 10.1186/2051-1426-2-S2-P14

**Published:** 2014-03-12

**Authors:** G Fey, TA Braciak, S Wildenhain, CC Roskopf, C Schiller, N Fenn, IA Schubert, U Jacob, KP Hopfner, FS Oduncu

**Affiliations:** 1 Institute for Microbiology, Biology, Erlangen, Germany; 2 Klinikum der Universitaet München, Haemato-Oncology MED IV, Munich, Germany; 3 Ludwig Maximilians Universität, Gene Center, Munich, Germany; 4 SpectraMab GmbH, Management, Munich, Germany

## Background

Current chemotherapy of acute myeloid leukaemia (AML) is limited by systemic toxicity. New agents are needed with increased selectivity for malignant cells and reduced toxicity.

## Materials and methods

Triplebodies are a new class of antibody derivatives developed by our team, which consist of 3 antigen-binding domains carried in a single polypeptide chain. The distal domains bind 2 targets on the same cancer cell, the central domain binds a trigger molecule on an effector cell. If the 2 targets on the cancer cell are different, the triplebody is said to be 'dual-targeting'. The key new capability resulting from dual-targeting is the elimination of cancer cells with 'increased selectivity' (Schubert 2013; MAbs. PMID 24135631).

Here the dual-targeting triplebody 33-16-123 (SPM-2) was developed and tested in redirected lysis assays *in vitro*. It carries binding domains for the surface antigens CD33 and CD123 on AML cells and for CD16, the Fc gamma RIII receptor on Natural Killer (NK) cells. This pair of target antigens is expressed on the blasts of a majority of AML patients and is present in increased surface density on AML-leukaemia stem cells (LSCs) relative to bulk AML cells and healthy hematopoietic stem cells. This combination offers the possibility of preferential targeting of AML-LSCs, and thus of minimal residual disease (MRD) cells.

Human AML cell-lines and primary cells freshly drawn from AML patients were used in redirected lysis experiments. Target cells were labeled with Calcein AM. Effector cells were either *ex vivo* expanded mononuclear cells from healthy donors, or patient's autologous NK cells enriched by immunomagnetic beads. Reactions proceeded for 4 hrs, and specific lysis was measured by release of fluorescent Calcein using an ELISA plate reader. Multicolor cytofluorimetry with fluorescent-labeled antibodies was used in addition to study elimination of subsets of AML cells.

## Results

SPM-2 effectively eliminated human AML cell lines including MOLM-13, which are double-positive for CD33 and CD123, with an EC50 concentration in the range of 20-50 pM, as well as primary blasts from AML patients with various subtypes of AML with EC50 concentrations in the range of 50-100 pM. Less than 10% of myeloid cells from healthy donors enriched for CD33-positive cells were lysed by SPM-2 used in a 10 nM dose. Therefore, a clear therapeutic window appears to exist. For some patient samples SPM-2 also produced effective lysis of the CD34pos and the CD34pos CD38neg CD123pos subsets, which encompass the MRD cells.

## Conclusions

SPM-2 mediated efficient redirected lysis *in vitro* by NK-cells, both of established human AML-derived cell lines and of freshly drawn blasts from patients with various subtypes of AML. SPM-2 also mediated lysis of cellular subsets encompassing the AML-LSCs and thus of the MRD cells responsible for relapsed disease. The agent is promising for lysis of AML cells *in vivo* with increased selectivity and reduced systemic toxicity and is scheduled for clinical development.

